# Retropharyngeal Space Schwannoma: A Rare Entity

**Published:** 2017-11

**Authors:** Stefania Gallo, Francesco Bandi, Marco-Paolo Maffioli, Marco Giudice, Paolo Castelnuovo, Enrico Fazio, Apostolos Karligkiotis

**Affiliations:** 1 *Department of Otorhinolaryngology, University of Insubria, Varese. ASST dei Sette Laghi, Ospedale di Circolo e Fondazione Macchi.*; 2 *Department of Otorhinolaryngology, Ospedale Giovanni Borea, Sanremo.*

**Keywords:** Cervicotomic approach, Retropharyngeal space, Schwannoma, Transoral approach, Transmandibular approach

## Abstract

**Introduction:**

Retropharyngeal space schwannomas are rare entities. About 20-45% of schwannomas occur in the head and neck region; however, they represent less than 1% of all head and neck tumors.

**Case Report:**

We present the case of a 36-year-old woman complaining of dysphagia caused by a large schwannoma arising in the posterior pharyngeal wall with remarkable reduction of the oropharyngeal space. The tumor was resected through a combined transoral and cervicotomic transmandibular approach due to its dimension. No recurrence was observed after a two-year follow up. This case represents the thirteenth case reported in international literature.

**Conclusion:**

Preoperative settings for rare tumors such as retropharyngeal schwannomas should include radiological investigations and preoperative biopsy. In order to obtain a successful result in terms of radicality, a combined surgical approach may be necessary to completely control the extension of the lesion.

## Introduction

Schwannomas are uncommon benign neurogenic tumors, arising from the sheath of the Schwann cells. They may originate from any neural structure in the head and neck region (1). Most schwannomas arise from the parapharyngeal space, including those associated with neurofibromatosis (2). However, since the retropharyngeal space includes less anatomical structures, tumors arising around this region are extremely rare (3). The standard treatment of schwannomas is surgical resection (4). The identification of the nerve’s origin and the ability to differentiate among various pathological processes that occur in the retro and parapharyngeal spaces are important for the preoperative planning of the surgical approach. To our knowledge only 13 cases of retropharyngeal schwannomas (RS) have been previously published, including the present case (5-16). 

## Case Report

**Fig 1:**
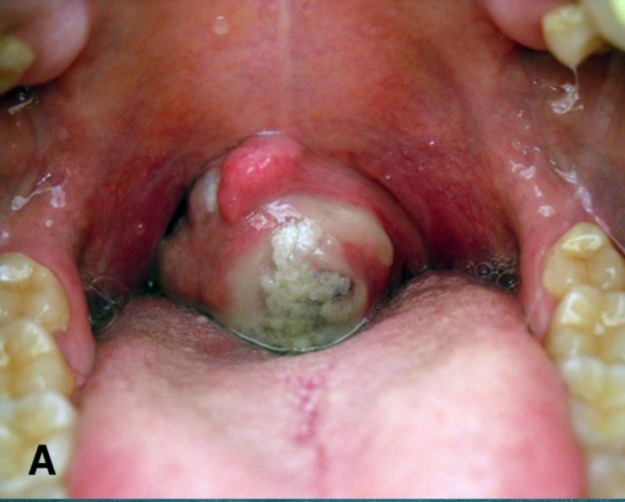
Intraoperative examination shows an anterior bulging of the left posterior pharyngeal wall due to the presence of a large mass with central mucosal ulceration and remarkable reduction of the oropharyngeal space.

A 36-year-old woman was admitted to our department with a 3-year history of a foreign body perception in the throat with gradually progressing dysphagia, associated with occasional odynophagia. A significant weight loss (6 kg) in the previous 2 months occurred. Physical examination revealed an anterior bulging of the left posterior pharyngeal wall due to the presence of a large, smooth mass, with central mucosal ulceration, causing a remarkable reduction of the oropharyngeal space (Fig. 1a).

**Fig 2:**
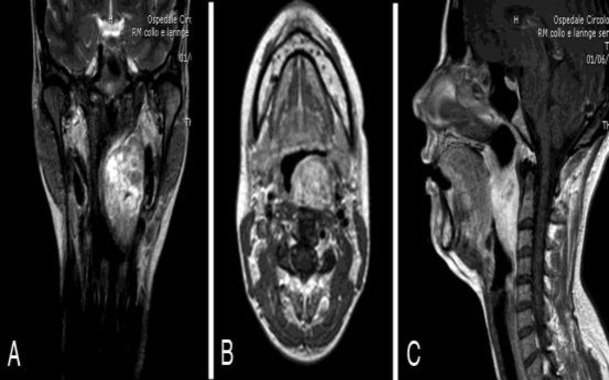
Preoperative MR shows a large mass arising in the left retropharyngeal space with bright signal intensity on T2-weighted images in coronal view (A) and a strong enhancement after gadolinium administration on T1-weighted images in axial view (B) and sagittal view (C).

The endoscopic evaluation pointed out the extension of the mass from below the left Eustachian tube to the omolateral piriform sinus. Nasal cavities were free and morphology and functionality of the larynx were preserved. Neck examination was otherwise normal. Magnetic resonance (MR) showed a well-defined encapsulated tumor in the left retropharyngeal space (size 70 mm x 40 mm x 30 mm), with high signal intensity on T2-weighted sequences and isointensity on T1-weighted, which was strongly enhanced by gadolinium administration (Fig. 2). 

The mass, with predominant submucosal development, was extending into the retropharyngeal space from the nasopharynx to the hypopharynx. 

An angio-CT scan was performed before the main surgical procedure, to evaluate the relationship between the tumor and the main vascular structures of the neck. A posterior displacement of the external carotid artery with an apparently preserved dissection plane was observe. The internal carotid artery was not in contact with the lesion. 

**Fig 3:**
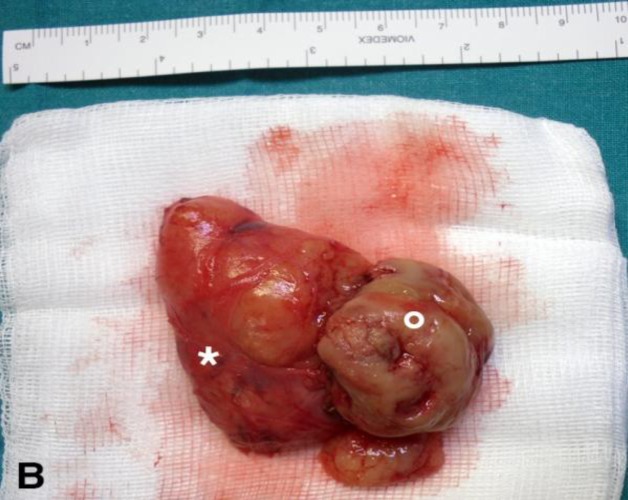
Gross image of the surgical resected specimen with clear evidence of the intraoral part (white circle) and the cervical part (white star) of the lesion

A biopsy, was performed under general anesthesia in order to define the lesion’s nature, which was diagnosed as a schwannoma. The patient underwent a combined transoral and cervicotomic transmandibular approach. The tumor possessed a plurilobulated aspect, bulging out in the oropharynx and developing in the retropharyngeal depth. Dissection was performed accurately and the lesion was removed in en-block throughout the oral cavity (Fig. 3). 

Tracheotomy was performed temporarily in order to prevent eventual postoperative dyspnea and was closed in 8 days later. Tumor size was 60 x 55 x 32 mm and the cross-section showed a “fish flesh” soft tan appearance. 

Histologically, the tumor was highlighted by a fibrous capsule composed of spindle cells with focal palisading of the nuclei arranged in short bundles and fascicles, which raise suspicion for schwannoma. The patient was fed through a feeding tube for 7 days and was dismissed 12 days after surgery, with a normal diet. No recurrence was observed during the two years of follow-up.

## Discussion

Schwannomas are rare, slow-growing, encapsulated, and submucosal tumors, more frequent in females between between the third and seventh decades. About 25-45% of schwannomas occur in the head and neck region and they may arise from some cranial nerves, especially from the IX^th^, X^th^, XI^th^, XII^th^ cranial nerves or the cervical sympathetic chain (17). The parapharyngeal space is the most common site of occurrence, followed by the oral cavity, nasal cavity, and paranasal sinuses (2). RS are extremely rare and our review of the literature highlighted only 12 additional cases arising in this region (Table.1) (5-16). In contrast with tumors arising in the parapharyngeal space, which are often asymptomatic, tumors arising in the retropharyngeal space produce signs and symptoms related to the mass effect with dysphagia being the most frequent, followed by dyspnea, and impair phonation.

**Table 1:** Summary of cases of retropharyngeal space schwannomas reported in international literature, focusing on dimensions of the lesions and relative surgical approaches

**Author**	**Year**	**N°**	**Age**	**Site**	**Dimension**	**Treatment**
André et al (5)	1977	1	45	PPW	ns	Local excision
Triaridis et al (6)	1987	1	21	PPW	20x30 mm	Trans-oral
Singh et al (7)	1995	1	36	PPW	60x50 mm	Trans-oral
Haraguchi et al (8)	1996	1	60	PPW	ns	Trans-oral
Moore et al (9)	1997	1	39	PPW	40x45 mm	Trans-oral
Torre et al (10)	1999	1	81	HPX	ns	Trans-oral
Thurnher et al (11)	2002	1	75	OPX	30x30 mm	Trans-oral
Huang et al (12)	2002	1	24	PPW	30x20 mm	Trans-oral
Hsieh et al (13)	2006	1	44	PPW	35x30x25 mm	Trans-oral
Kumagai et al (14)	2006	1	24	PPW	37x18x16 mm	Trans-oral
Jovanovic et al (15)	2008	1	59	PPW	20 mm	Trans-cervical
Jovanovic et al (16)	2008	1	38	PPW	250x60x40 mm	Trans-cervical
Present case	2012	1	36	PPW	60x55x32 mm	Trans-cervical/Trans-oral

PPW, posterior pharyngeal wall; HPX, hypopharynx; OPX, oropharynx; ns, not specified

Diagnosis is challenging since in the retropharyngeal space, which contains essentially fat and lymph nodes, the most frequent diseases are metastasis from primary head and neck cancer and lipomas, neuroblastomas and pleomorphic adenomas (13). MR with gadolinium enhancement is particularly helpful in defining schwannomas. Usually these tumors have a low signal intensity on T1-weighted sequences, a high signal intensity on T2-weighted sequences, and a strong enhancement after gadolinium administration. No flow voids are seen in these tumors (17). However, none of these characteristics are specific for a clear diagnosis. A biopsy might be considered if the radiological investigations exclude the vascular nature of the lesion and the site is reachable through a minimally invasive approach (15).

The determination of the nerve of origin with imaging studies, before the surgical exploration, still remains difficult in many cases. Various authors described the criteria for the determination of the nerve’s origin in the parapharyngeal space on CT or MR findings (4). However, in our case the retropharyngeal location of the tumor was suspicious for a small nerve supplying the pharynx (peripheral plexus) (18).

The treatment of schwannoma is based on a radical surgical resection with the preservation of nerve function, except when the size or the location of the tumor does not allow for a complete excision, forcing only a partial removal or sacrifice of the nerve.

However, in view of the fact that schwannomas are separated from nerve fibers by a fibrous capsule, in contrast to neurofibromas in which the nerve is an integral part of the neoplasm and must be sacrificed in order to excise the tumor, in most cases it possible to enucleate the tumor with the preservation of the nerve (19). 

Many surgical procedures have been described, depending on the site and the size of the tumor, such as trans-oral, trans-cervical and trans-parotid approaches, variously combined (3).

## Conclusion

Retropharyngeal schwannomas are uncommon diseases with a challenging diagnosis. Radiological examinations are mandatory in order to define the non-invasive and avascular nature of the lesion. A preoperative biopsy has a diagnostic significance and should be taken into account. In order to obtain a successful result in terms of radicality, a combined surgical approach may be necessary to completely control the extension of the lesion.
